# The natural environment: a critical missing link in national action plans on antimicrobial resistance

**DOI:** 10.2471/BLT.18.210898

**Published:** 2018-10-10

**Authors:** Graziella Iossa, Piran CL White

**Affiliations:** a School of Life Sciences, Joseph Banks Laboratories, University of Lincoln, Lincoln LN6 7TS, England.; b Department of Environment and Geography, University of York, York, England.

Antimicrobial resistance, that is, the ability of a microorganism to stop an antimicrobial from working against it, is one of the most important global health challenges. Antimicrobial resistance is projected to become the leading cause of death worldwide, claiming an estimated 10 million lives a year by 2050, primarily in low- and middle-income countries.^1^ In 2015, the World Health Assembly adopted a global action plan on antimicrobial resistance underpinned by the One Health approach.^2^ One Health seeks to improve health and well-being through the integrated management of disease risks at the interface between humans, animals and the natural environment. Efforts are based on closer collaboration among individual disciplines and a cross-sectoral approach to research, surveillance and response.^3^ The natural or biophysical environment here includes all living (biotic) and non-living (abiotic) factors affecting the survival of all organisms, including humans, at the individual, population, community or ecosystem level. In this context, an ecosystem refers to a community of plants, animals and microorganisms that live, feed, reproduce and interact within the same area or environment. Antimicrobial resistance connects human health to the health of ecosystems and the natural environment. With the global action plan on antimicrobial resistance, Member States agreed to develop individual national action plans on antimicrobial resistance by May 2017.^2^^,^^4^ Here we present an analysis of the extent to which the national action plans developed after 2015 have been successful in integrating the natural environment within a One Health approach. We found that the action plans feature human and animal health prominently, but that most do not specifically incorporate the natural environment, and hence fall short of achieving a genuine One Health approach. Given the key role of the natural environment in contributing to antimicrobial resistance, we call for greater integration of the natural environment in existing and new national action plans, so that we can maximize our chances of finding holistic and sustainable solutions to this global health threat.

## Resistance in the environment

Antimicrobial resistance develops in, and is maintained and transmitted across humans, animals and the natural environment. Resistance is an ancient and naturally occurring phenomenon, but anthropogenic antibiotic use since the 1930s has been linked to the increasing occurrence of antimicrobial-resistant microorganisms, particularly bacteria.^5^^,^^6^ The natural environment presents a transmission route and a reservoir for resistant microorganisms and plays a significant role in the development of, and response to, antimicrobial resistance.^7^ Antimicrobial use in the livestock industry is driving the selection of resistant bacteria in farm animals, and while the causal link between antimicrobial use in livestock and the selection of resistance in humans is difficult to establish, there is evidence of an association.^8^ Resistant bacteria of animal origin can be transmitted to humans directly via food or indirectly via the natural environment. Moreover, wastewater treatment plants, manure from farm animals and spill-over from the pharmaceutical production industry contribute to an environmental reservoir of resistance.^7^ The natural environment should therefore be a key consideration in understanding and developing strategies to control antimicrobial resistance, as acknowledged through the One Health approach.^9^

## National action plans

One of the key guiding principles of the national plans is to use a One Health approach and multisectoral coordination. This principle gives the various collaborating organizations equal status and emphasizes a sense of ownership of the plan.^4^ As of May 2018, 56 Member States (29.0%) out of 194 have published national action plans, of which 21 have been published since 2015.

We analysed the 18 national action plans published in English between 2015 and October 2017 (Box 1) to determine the extent to which the plans integrated the natural environment within a One Health approach. We used content analysis^10^ by constructing a set of categories: (i) One Health (inclusion of the term itself, related terms or no reference); (ii) human-animal-agriculture (agriculture appeared to be used as synonymous for natural environment; inclusion of these keywords was linked to One Health, but with no specific mention of One Health, related terms or no reference); (iii) environment (inclusion of the term itself, related terms or no reference); and (iv) human-animal health (inclusion of the term itself, related terms or no reference). The categories also identified the broader context in which those terms were used: in the introduction or problem definition of antimicrobial resistance; in the plans’ strategic objectives; in the implementation; and/or in the evaluation process. As we were particularly interested in multisectoral approaches to tackling antimicrobial resistance, we also searched for evidence of the presence or absence of such approaches in the national action plans.

Box 1 **The national action plans on antimicrobial resistance considered in our analysis, by WHO Region**

**African Region**

United Republic of Tanzania (2017)

**Region of Americas**

Barbados (2017) and Canada (2017)

**Eastern Mediterranean Region**

Oman (2017)

**European Region**

Denmark (2017) and Sweden (2016)

**South-East Asia Region**

Bangladesh (2017), India (2017), Indonesia (2017), Maldives (2017), Nepal (2016), Sri Lanka (2017), Thailand (2016) and Timor-Leste (2017)

**Western Pacific Region**

China (2016), Japan (2016), Mongolia (2017) and New Zealand (2017)

Ten out of 18 action plans followed the World Health Organization (WHO) guidelines and identified five objectives in line with the global action plan on antimicrobial resistance: (i) improve awareness and understanding of antimicrobial resistance through effective communication, education and training; (ii) strengthen the knowledge and evidence base through surveillance and research; (iii) reduce the incidence of infection through effective sanitation, hygiene and infection prevention measures; (iv) optimize the use of antimicrobial medicines in human and animal health; and (v) develop the economic case for sustainable investment that takes account of the needs of all countries, and increase investment in new medicines, diagnostic tools, vaccines and other interventions.^2^^,^^4^ The eight remaining action plans identified further country-specific objectives. Encouragingly, a further objective was to establish governance and a multisectoral approach to combat antimicrobial resistance.

**Fig. 1:**
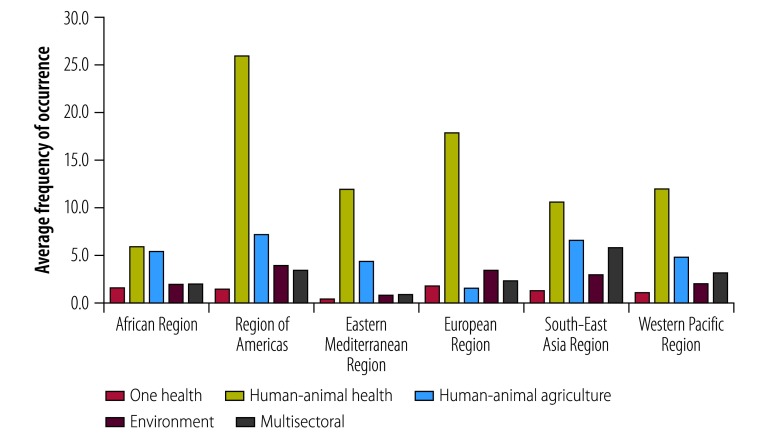
Content analysis of national action plans on antimicrobial resistance published after 2015

Note: Content analysis (average frequency of occurrence) of the search terms around One Health, human-animal health, human-animal-agriculture, environment and multisectoral engagement for 18 national action plans on antimicrobial resistance published after 2015.

The most frequent term across all action plans (Fig. 1) was that of human-animal health, which had an average frequency of occurrence of 14.1 in the text of the action plans. This was followed by human-animal-agriculture, which occurred an average of 5.1 times, and the environment, which occurred an average of 2.6 times. No plans included the terms ecology, ecologist or ecosystem. Ecosystem was mentioned in five plans but the context, for instance “clinical settings represent an ecosystem of high antimicrobial usage” or “the entire ecosystem of antimicrobial resistance,” suggests a non-ecological meaning. The term One Health occurred an average of 1.3 times in the plans and multisectoral coordination occurred an average of 3.0 times (Fig. 1). Across all plans, with regards to the context, a One Health approach was occasionally referenced in the introductory section and in the definition of antimicrobial resistance as a challenge. Twelve of the 18 plans also mentioned a One Health approach in their strategic objectives. However, this was mainly restricted to the need for an integrated surveillance system, with the acknowledgement of a current focus on human and animal health and an intention to integrate the natural environment in the future. Reference to a One Health approach was related mainly to multisectoral collaboration and to a whole-of-society engagement. In contrast, there was frequent reference to human-animal-agriculture terms in the strategic objectives, but these were not specifically linked to One Health.

Although most plans have been drafted by multisectoral coordination teams who acknowledge that One Health guides their approach, the predominance of topics around human-animal health and the scarcity of topics around the natural environment suggests that there are more representatives of human-animal health in the coordination teams. The reason for this could be twofold. First, we have an increasing understanding of resistance mechanisms in human-animal systems, alongside better developed surveillance methods, and longer-term collaborations among key agencies, such as WHO, the Food and Agriculture Organization of the United Nations (FAO) and the World Organisation for Animal Health (OIE).^7^^,^^9^ Second, the recent history of the One Health approach has stemmed from the need to confront emerging infectious diseases outbreaks. The development of the One Health approach has therefore so far has been primarily embraced by veterinarians and, to a certain extent, physicians.^3^ Currently, two of greatest challenges to ensure that One Health becomes embedded in governance are ensuring that greater emphasis is placed on the key role of the natural environment and ecosystems in contributing to antimicrobial resistance risk and management,^11^ and working to break down disciplinary and policy silos and embrace the changes needed to implement a genuinely interdisciplinary and cross-sectoral approach.^12^

## Concluding observations

Antimicrobial resistance is a complex multisectoral challenge. Some progress is being made towards embedding a One Health approach in national action plans on antimicrobial resistance. For example, the national plan of Sri Lanka provides an example of a cross-sectoral approach including expertise from rural economy, fisheries, aquatic sciences, veterinarians and public health, and the national plan of India includes an objective on reducing the environmental spread of antimicrobial resistance. However, our analysis suggests that the first set of national action plans have not been wholly successful in incorporating a One Health approach. The plans have prompted the establishment of multisectoral committees and, in some cases, governance. Yet, while human and animal health feature prominently, the natural environment has yet to be fully integrated into these plans. Since the natural environment is a key source and driver of antimicrobial resistance, as highlighted by the WHO/OIE/FAO Tripartite and by the global action plan on antimicrobial resistance,^2^ this is an important omission. More research on the ecology of antimicrobial resistance in the natural environment is needed, for example on the population dynamics of antimicrobial resistant genes and resistant microorganisms; the transmission dynamics of resistance including the role of wildlife;^13^ the role of biodiversity in enhancing or regulating resistance; the selection pressures operating on resistance; and the role of co-selective factors such as biocides and metals. Without the more specific integration of the natural environment into national action plans and future strategies, we risk jeopardizing many of our efforts to tackle the increasing threat posed by antimicrobial resistance.^7^
